# Purification, Detoxification, and Incineration Methods of Minerals and Metals in Traditional Medicine Formulations of Sri Lanka

**DOI:** 10.1155/2021/6634553

**Published:** 2021-01-15

**Authors:** Horadugoda Gamage Sujatha Pushpakanthi Hewageegana, Ayuma Uththami Hewageegana, Liyanage Dona Ashanthi Menuka Arawwawala

**Affiliations:** ^1^Department of Kayachikitsa, Institute of Indigenous Medicine, University of Colombo, Colombo, Sri Lanka; ^2^Faculty of Science, University of Colombo, Colombo, Sri Lanka; ^3^Industrial Technology Institute, Bauddhaloka Mawatha, Colombo 07, Sri Lanka

## Abstract

**Background:**

Herbo-mineral therapies are very popular in traditional medical systems and formulations consisting of specific minerals or metals or mixture of both and mixed with organic components derived from plants. Purification/detoxification or incineration procedures play an important role to detoxify these and metals and minerals.

**Objective:**

In the present review, an attempt was made to gather herbo-mineral formulations which are used commonly in traditional medicinal systems in Sri Lanka and recapitulate the purification/detoxification or incineration techniques.

**Method:**

Commonly used herbo-mineral formulations are collected from a text book of Vatikaaprakarana. However, the purification/detoxification and incineration techniques for all minerals/metals are not mentioned in Vatikaaprakarana, and these techniques were collected from journal articles published between 1^st^ January 2000 and 1^st^ June 2020 through searching PubMed (US National Library of Medicine, USA), Science Direct (RELX Group, Netherlands), and Semantic Scholar (Allen Institute for Artificial Intelligence, USA).

**Results:**

Ten herbo-mineral formulations were selected, and purification/detoxification or incineration techniques were described in brief for copper sulphate, aluminum sulfate, borex powder, sulphur, sodium chloride, cinnabar, arsenicals, realgar, orpiment, ammonium chloride, magnesium silicate, zinc, and mercury.

**Conclusion:**

The review has demonstrated different types of purification/detoxification or incineration techniques of minerals used in herbo-mineral preparations. In addition, there is an urgent need for comprehensive survey or evaluation to check whether purification/detoxification or incineration techniques of metals/minerals are practiced properly in the country.

## 1. Introduction

Sri Lanka has its own indigenous scheme of healing systems including Ayurveda, Siddha, Unani, and traditional medicine. Sri Lankan traditional medical system is based on a series of prescriptions handed from generation to generation over 3000 years [1]. Herbo-mineral therapies are very popular in traditional medical systems of Sri Lanka specially for diseases which are related to neuromuscular disorders, malnutrition, different types of fever, and worm and digestive tract disorders, and herbo-mineral formulations consist of specific minerals (e.g., mica, realgar (As_2_S_2_), orpiment (As_2_S_3_), chalk (CaCO_3_), salts (NaCl or KCl), magnetite (Fe_3_O_4_), pyrite (FeS_2_), etc.) [2] or metals (e.g., gold, copper, iron, zinc, mercury, etc.) [3] or mixture of both. In addition, some herbo-mineral formulations consist of plant/s along with minerals or metals or both. Herbo-mineral formulations are used in Rasayana therapy for several external and internal diseases [4]. It is claimed that herbo-minerals can become very less or nontoxic due to the purification and detoxification techniques during the drug preparation procedures. Many metals such as zinc [5, 6], copper [7, 8], and iron [9, 10] exhibit many therapeutic benefits in humans. However, excess amounts of these metals may exhibit toxic effects. Therefore, metals/minerals present in herbo-mineral formulations should be subjected to purification/detoxification and/or incineration procedures.

Most of raw materials (metals/minerals) are collected from earth and there is a chance for mixing of impurities, toxins, and heterogeneous and unwanted substances to a large extent. Hence, these are subjected to various methods of purification and detoxification techniques (Shodhana) and incineration (*Marana*) to make them competent for medicinal use. Both purification/detoxification and incineration techniques are indicated to induce certain qualities, which are essential for making sure of the safe and easy assimilation of the material in the living body.

Most of the herbo-mineral recipes mentioned in traditional medicine in Sri Lanka are in the form of paste (kalka) or in pills (guli). Buddharaja kalka and Desadun kallka are used in the form of paste. Seetarama guliya, Koladavunde, Suranvidura guliya, Diyatarama guliya, and Krimiraja guliya are the common form of pills. These pastes or pills contain the minerals such as thurisi (copper sulphate), savindalunu (rock salt), sinakkaram (aluminium sulphate), pushkara (borax sodium biborate), nellikka gendagam (sulpher sublimatum), sivanguru (kaolinumbolerubja (china clay)), sadilingam (hydragyrisulpidum), haritala (trisulfide of arsenic), manosila (arsenigralar), galmada (rock alum), galnahara (asbestos), black salt (suwasa lunu), swarna makshika (ferri sulphuratum), and shilajathu (asphaltum/asphalt mineral). In this review, an attempt was made to gather the herbo-mineral formulations which are used commonly in traditional medicinal systems in Sri Lanka and display the purification/detoxification and incineration techniques.

## 2. Materials and Methods

In Sri Lanka, traditional herbo-mineral formulations are documented in the textbook known as Vatikaaprakarana [11]. In the present survey, some of the herbo-mineral preparations described in Vatikaaprakarana are costly and the processes of purification/detoxification and incineration techniques need much effort. Total numbers of 143 formulations (both herbal and herbo-mineral) are found in Vatikaaprakarana. In addition, all the purification/detoxification and incineration techniques for herbo-minerals are not mentioned in Vatikaaprakarana. Therefore, commonly used purification/detoxification and incineration techniques are followed whenever required. In the present survey, purification/detoxification and incineration techniques were collected from journal articles published between 1^st^ January 2000 and 1^st^ June 2020 through searching PubMed (US National Library of Medicine, USA), Science Direct (RELX Group, Netherlands), and Sementic Scholar (Allen Institute for Artificial Intelligence, USA). The keywords are “herbo-minerals and toxicity” and “herbo-minerals and purification and incineration”. From a total number of 165 results, 40 were excluded due to duplication or being irrelevant for the title. The frequently used traditional herbo-mineral formulations used in Sri Lanka are given in Table 1.

## 3. Purification/Detoxification and Incineration Techniques

Purification/detoxification and incineration of minerals and metals are very essential to avoid unwanted hazards caused by herbo-minerals.

### 3.1. Copper Sulphate-Palmanikkam

#### 3.1.1. Method 1

Seven hundred and fifty grams of crude copper sulphate is dissolved in water, filtered through a cloth and dried in shade to remove external impurities. Dried copper sulphate is put into a mortar and 350 ml of *Citrus acida* Roxb juice is poured to make it sufficiently wet. Then, content is triturated manually using the paste under constant pressure for 6 h. The paste is allowed to dry and then preserved. Approximately 1% weight loss can be observed after purification of copper sulphate [12].

#### 3.1.2. Method 2

Copper sulphate is converted into a powder form using “*khalva yantra”* (it is an instrument that can made up of good quality of stone useful for trituration and levigation). After that, copper sulphate powder is collected on a three-layered cloth and closed firmly by a tight knot. Subsequently, cow urine is collected and filtered to a steel vessel through a filter paper. Then, 8 L of cow urine is added to a special vessel called “*dolayantra*” and copper sulphate which is tightened with cloth which is dipped in three fingers above from the bottom of vessel. Then, “*dolayantra*” is kept on a stove and exposed to mild heat and the cow urine is left to boil for 9 hours. When the level of cow urine is decreased, extra 500 ml cow urine is added again to it. After completion, leave it to cool and allow it to settle down for re-crystallization for 24 hours under room temperature. The crystals of copper sulphate are formed at bottom of the *“dolayantra”* and supernatant liquid is removed. Copper sulphate crystals are collected and allowed to dry in shade [13].

### 3.2. Aluminum Sulfate/X Al(SO_4_)_2_12H_2_O-Alum (Phitkari)

Formulation is X Al(SO_4_)_2_12H_2_O, where X is a monovalent cation such as potassium or ammonium. Alum or phitkari is a transparent salt-like substance that is used in cooking as well as for medicinal purposes. In Ayurveda, alum (phitkari) is used in the form of Bhasma (Pure Ash) called Sphatika Bhasma.

Method: impure alum is dissolved in water and the insoluble impurities are removed through filtration. Then, the solution is concentrated and kept in a container with cooled water [14, 15].

### 3.3. Borax Powder (Sodium Borate)-Puskara/Tankana

Puskara or tankana kshra ([Na_2_B_4_O_5_(OH)_4_].8H_2_O) is composed of boric acid and soda. Chemical name of borax is sodium tetraborate decahydrate. Scientific investigations have proven the antimicrobial [16] and anti-inflammatory activities [17] and wound healing [18] properties of borax powder.

#### 3.3.1. Method 1

Borax powder is put into a clean and dry *“khalva yantra”* and crushed well to prepare powder. This powder is added to a clean earthen pot and heated under moderate heat to maximum heat until all the water content is completely evaporated to obtain borax powder. This procedure should be repeated thrice to obtained pure borax and approximately 50% weight loss can be observed [19, 20].

#### 3.3.2. Method 2

Raw borax is powdered, then put to a hot iron pot, and stirred till it intumesces. Finally, borax is taken out and ground to become fine powder [21].

#### 3.3.3. Method 3

Borax is dissolved in water, strained through a cloth, and evaporated to dryness [22].

### 3.4. Sulphur-Sulphur Sublimatum/Gandhaka

Sulphur sublimatum or gandhaka is one of the members of Upa Rasa (one of the two groups of alchemical mineral agents, according to the Rasashashtra) by Rasa Vagbhata found and described in the Bible as brimstone [23]. The sulphur is extensively used in herbo-mineral preparations especially in various mercurial operations. There are several methods, which have been mentioned in different texts, for the Shodhana of gandhaka. It can purify blood, support for healthy digestion, and prevent build-up of toxic substances [24]. Gandhaka contains two kinds of impurities: (a) stone powder or clay (b) As, Pb, or both [25]. Therefore, it is important to remove physical and chemical impurities in sulphur.

#### 3.4.1. Method 1

The commonest method consists of heating of sulphur with cows' ghee into its melting stage and filtering into a vessel containing cow milk through a cloth. Finally, it is washed with hot water and the whole procedure should be repeated three to seven times using fresh ghee and fresh cow milk. In the present process, chemical impurities bind with ghee and physical impurities are removed while filtering through the cloth [26, 27].

#### 3.4.2. Method 2

Crude sulphur (100 g) is powdered and placed on an iron/steal spoon containing 25 ml of cow's ghee and subjected to mild heat while stirring the content using a glass rod. After all the solid particles are melted, the content is filtered through a cloth into a vessel containing freshly prepared *Eclipta prostrate* L. (Bhringaraja) juice. Sulphur becomes solidified with presence of *Eclipta prostrate* juice. Then, collected sulphur is washed with hot water to remove the traces of ghee and juice. This purification procedure should be repeated at least six times using the fresh juice of *Eclipta prostrate* [28].

#### 3.4.3. Method 3

Cow milk (2 L) and ghee (150 ml) are added to an earthen vessel with wide mouth and covered by a cloth and tied with an iron wire. Approximately 500 g of coarse powder of gandhaka is applied on the inner surface of the cloth which covers the lower earthen vessel containing cow milk and ghee. Then, the lower pot is covered with another earthen vessel by placing in up-down position. The edges of both earthen vessels are sealed with a cloth applied with Fuller's earth and allowed to dry and kept inside a pit (1.5 feet from the upper surface of the earth) in a way that the brim of the vessel should be at ground level. Then, cow dung cakes are kept on the brim of the vessel and fire is set and the sulphur is allowed to flow down into the vessel containing cow milk and ghee. Once it is cooled, whole vessel is taken out from the pit and opened. The purified sulphur is collected and washed with hot water to remove the traces of ghee and shade dried [29].

#### 3.4.4. Method 4

The sulphur purification steps for heating, melting, and filtering are the same as method 2 [28]. In brief, *Millettia pinnata* L. oil (Karanja oil, 25 ml) and *Ricinus communis* oil (Erand oil, 25 ml) are mixed together and used instead of ghee. In addition, goat milk and *Datura metelis* L. (Dhattura patra) juice are used instead of *Eclipta prostrate* juice. Sulphur is filtered through the cloth into a vessel containing goat milk and *Datura metelis* juice thrice, respectively. Finally, sulphur is washed and dried [28].

### 3.5. Sahindava Lavana-Sodium Chloride/Rock Salt/Bay Salt

There are five types of lavana commonly used in treatments. Among the lavana, saindhava lavana is the best and considered as sodium chloride/rock salt/bay salt chemically. Rock salt is the purest form of salt, which is free from environmental pollutants and chemical components and does not require a refining process [30]. Rock salt is used to cure several disorders and ailments including rheumatic pains, herpes, inflammation, common cold, and cough [31].

### 3.6. Cinnabar (Hydrargyri-HgS)

Sadilingam or hingula is an important drug in Ayurveda which is used as single remedy or as an ingredient in various herbo-mineral preparations. By most of the Ayurveda Rasa Shastra classics, it is grouped under Sadharana Rasa Varga. Hingula is the prime source of mercury. Chemically, it consists of mercury (86%) and sulphur (13.5%), with molecular formula HgS, called red mercury sulphide. Purified mercury is used in the treatment of eye diseases, disorders in liver and pancreas, rheumatoid arthritis, fever, and skin disorders [32].

#### 3.6.1. Method 1

A paste of cinnabar is prepared by triturating with *Zingiber officinale* Roscoe. (Aardraka Swarasa) or *Citrus limon* L. (Nimbu Swarasa) or *Allium sativa* L. (Lakucha Swarasa) and applying on the inner surface of the upper pot. The lower pot filled with sufficient amount of water is buried in the earth with neck above. Then, the pot that cinnabar paste is applied to is placed in an up-down position and the edges of both earthen vessels are sealed. Then, cow dung cakes are kept on the top of the upper vessel and fire is set and allowed to heat for three hours. The purified mercury from base of upper pot gradually collected trickles down and gets collected at the base of the lower pot. After 24 h, the lower pot is taken out with mercury [32].

#### 3.6.2. Method 2

Unpurified hingula is smashed into powder in mortar triturated with ginger juice (*Zingiber officinale*) seven times using a pestle. After seven times trituration, hingula turns into a crystallized powder form bearing an acidic pH. Then, crystallized powder is washed with hot water until acidity is completely removed and dried in sun light [33].

#### 3.6.3. Method 3

Hingula is soaked in *Citrus limon* juice until the colour of the powder becomes dark red, then it is washed with water at least seven times [34].

### 3.7. Arsenicals

*α*-As_2_S_2_ is structurally identical to realgar (*Manashila*) among the three distinct As_4_S_4_ polymorphs [35] and identified as a promising drug for cancer treatments [36, 37]. Realgar has been used as an ingredient in Chinese Traditional Medicine for many centuries and some of the Realgar contained in *Hong Ling San, Sha Yao, Qi Zhen Wan*, etc. [38]. Other two polymorphs are orpiment (haritala) and white arsenic (gouripasana) with chemical structures of As_2_S_3_ and As_2_O_3_, respectively [39]. Some of the therapeutic uses of three As_4_S_4_ polymorphs are listed in Table 2.

Arsenic is listed as the most hazardous element by Agency for Toxic Substances and Diseases Registry (ATSDR) among the top 20 hazardous substances [41]. Therefore, purification is essential.

#### 3.7.1. Realgar (Manashila, Arsenic Disulphide-*α*-As_2_S_2_)

Realgar is used only in purified and detoxified condition for the therapeutic purposes as internal as well as external medicine. The most of the formulation of Manahshila is used for external application, whereas fine powders of these processed minerals are used for both external and internal applications. Purification of realgar is generally carried out by trituration with *Sesbania grandiflora* L. (Agastya), *Zingiber officinale* (Ardraka) (Rosc.), *Citrus medica* (Bijaura Nimbu), *Sesbania sesban* L. (Jayanti), and *Eclipta alba* L. (Bhringaraja) juice [42].

*(1) Method 1*. Realgar is placed in a mortar and mixed with the *Zingeber officinalis* juice and triturated with a pestle manually until the material in the mortar dries. This is repeated seven times with fresh *Zingeber officinalis* juice. Finally, purified realgar is shade-dried and powdered [43].

*(2) Method 2*. Realgar is triturated to 120 mesh size for particle size reduction. Detoxification of realgar (400 g) is done by boiling it in *Ecipta alba* juice, *Sesbania grandiflora* juice, *Sesbania sesban* juice, and *Zingiber officinale* juice sequentially, each for 12 hours in Dolayantra. Detoxified realgar is then washed with *Kanjika* (sour liquid prepared with of rice grain, etc.) and kept in airtight container after drying [44].

#### 3.7.2. Orpiment (Haratala-As_2_S_3_)

*(1) Method 1*. Orpiment is steamed (Swedana) followed by submerging the drug in liquid media for a whole night and during day time it is to be dried under sun light (Bhavana) or both [45]. The liquids like Tilaksarajala, Salmali mula Kwatha, lime juice, and Balamula Kwatha are also used by one or two Acharyas for the purification of orpiment either for Swedana or Bhavana. Swedana-Haritala churna should be kept in a pottali and this pottali should be placed in dolayantra containing various types of liquids. The dolayantra should be subjected to fire for given time [46].

*(2) Method 2*. Haratala is powdered and subjected to Bhavana for twenty-one days with the juice of *Ficus religiosa* L. (Asvattha). Using a clean mortar, haratala is made into a ball and kept in a vessel where one half is filled with ashes. The vessel is closed with a basin and subjected to incineration for 12 h [47].

#### 3.7.3. White Arsenic (Gouripasana-As_2_O_3_)

It is a crystalline or amorphous substance. When heating with intense heat, it evaporates and gives garlic oduor [48].

*(1) Method 1*. White Arsenic is kept inside a large fruit of *Momordica charantia* L. (Bitter gourd), then tied well, and subjected to steam *Dolyantra* (hanged in a big pot which is filled with water) for three hours. This process purifies the white arsenic [49].

*(2) Method 2*. White arsenic is kept inside a large fruit of *Momordica charantia* and tied well and subjected to steam in *Dolyantra* (hanged in a big pot which is filled with borex water or cow milk) for 24 hours [50].

Steaming of bitter gourd (filled with Gouripashana) is done in borex water (tankana jala) or cow's milk (godugdha) with the help of dolayantra, for one day. Cow's milk or borex water purifies the gouripashana [51].

*(3) Method 3*. Take purified (Shuddha) gouripashana in a mud pot. Put 15 g of Goat's milk and cover by a cloth. Place this in a pit and cover it by using mud by the width of middle phalanges of middle finger. Then, ignite using dried cow-dung as the fuel. This procedure is repeated 21 times. For each burning, add 56 ml goat's milk and finally get purified yellow red gouripasana [52, 53].

### 3.8. Sal-Ammoniac/Ammonium Chloride-NH_4_Cl-Navasadara

It is a crystalline, inorganic salt, white in colour and highly soluble in water [54].

#### 3.8.1. Method 1

Water (three parts) is added to navasaran (one part), stirred well, and filtered into a vessel using a cloth. Then, the vessel is heated until the solution is dried [55].

#### 3.8.2. Method 2

Water is added to navasaran, stirred well, and filtered to a pan and subjected to dry conditions in sun light [56].

### 3.9. Magnesium Silicate (Asbestos)-Gal Nahara

#### 3.9.1. Method

Boil magnesium silicate using Buffalo urine for three hours. Then, wash it and dry properly [14].

### 3.10. Rock Alum-Gal Mada

No purification method is mentioned in texts though these ingredients are commonly mentioned in traditional recipes. Further, according to Ayurvrda Pharmacopoeia in Sri Lanka [57], the materials gal mada and gal nahara can be used for medicinal preparations after removing the dirty particles.

### 3.11. Zinc Oxide-Ridi Thuththam

Zinc is a trace element and plays a vital role in all physiological processes in human. It has been introduced as a drug in the prevention and treatments of diseases for the last two decades. The Ayurvedic physicians have practiced both oral and topical applications of zinc after purification and calcification before 14th century A.D.

Rasaka or Kharpara (zinc ore or zinc carbonate), Yasada (zinc metal), Puspanjana (zinc oxide), and Pittala (brass) are zinc-containing minerals used as therapeutic agents in Ayurveda [58].

#### 3.11.1. Method 1

Raw *Yashada* pieces are heated in an iron ladle till they melt completely and then poured into a mixture of sour gruel, butter milk, decoction of *Dolichus biflorus*, cow's urine, and *oil* of *Sesamum indicum.* This is repeated 3 times in each liquid medium, in the same order [59].

#### 3.11.2. Method 2

Five hundred grams of zinc granules is kept in iron utensil and placed on a gas burner. Zinc granules are heated till they melt. The molten mass is poured in a container containing milk (200 ml). This exercise is repeated 21 times [60].

### 3.12. Mercury-Parada

Mercury (Parada) has many synonyms. Mercury is the best among all medicines which is effective in low dose and fast acting in the eradication of disease without causing anorexia or other side effects. Mercury in the form of one of its common ores, cinnabar, is used in various traditional medicines, especially in traditional Chinese and Indian medicine [57].

Mercury also undergoes extensive detoxification procedures before being used in medical formulations. Mercury obtained by all these procedures is an inorganic form of mercury (mainly sulphides). Studies have also validated this fact and showed that mercury used in Ayurveda is of inorganic form. Moreover, inorganic mercury does not cross the blood-brain barrier as well as placental barrier [58].

#### 3.12.1. Method 1

Equal quantities of mercury (500 g) and Sudha churna (limestone-500 g) are taken into a mortar and triturated for 36 hours (3 hours for 12 days). Mercury is collected from limestone filtered through a cloth. The remaining mercury is obtained by washing it with warm water. The wet powder of limestone is allowed to dry. After drying of these carefully, mercury is collected from these trays. Then, equal quantity of Lashuna Kalka 439 g (paste of garlic) is added to the obtained mercury and half the quantity of saindhava lavana (219.5 g) is added and triturated for 8 hours. Washing of garlic paste is done with lukewarm water. The salt present in it dissolves in water leaving behind the garlic paste from which again mercury can be collected. Once again, drying of the remaining garlic paste into trays is done for 6 days and then triturated into fine powder and filtered through cloth to obtain the remaining mercury from the garlic paste to avoid the loss of mercury. The collected mercury is known as purified mercury [59].

#### 3.12.2. Method 2

Mercury is taken with *Piper betel* L. (Nagavalli svarasa, 50 ml), *Zingiber officinalae* (Ardraka svarasa, 50 ml), and Trikshara (Yavakshara, Sarjika kshara and Tankana kshara, each of 50 g) in a clean *Khalva Yantra*. The above-said materials are rubbed in *Khalva Yantra* for eight hours per day for three days. The obtained material is washed and poured out with the help of lukewarm water several times until we get the pure mercury [57].

#### 3.12.3. Method 3

Mercury is roasted in a covered crucible with asafetida (dried latex exuded from *Ficus oppositifolia* Roxb) [61].

#### 3.12.4. Method 4

The seeds of *Achyranthes aspera* L and *Ricinus communis* L are pounded together and mercury is placed inside the powder. Then, the whole mass is subjected for incineration [61].

### 3.13. Safety Studies on Herbo-Minerals

Literature on evaluation of toxicity levels of metals/minerals in herbo-mineral preparations after purification/detoxification or incineration is limited. Some experiments were carried out to determine the overall toxicity of herbo-minerals using animal experiments in terms of hepatotoxicity, hemototoxicity, renal toxicity, etc. However, purification/detoxification or incineration techniques of herbo-minerals were not properly documented in those studies. Thus, one example is cited here in order to get an idea about toxicity of herbo-minerals. Doddamani and co-workers [62] found that Tribhuvan Keerthi rasa (contains cinnabar), Swasakutara rasa (contains mercury and sulphur), Smritisagara rasa (contains orpiment), Sutashekara rasa (contains mercury), Lashunadi vati (contains sulphur), and Agnitundi vati and Arogya vardini vati (contain both mercury and sulphur) did not affect kidney functions in terms of urea and creatinine levels. More than one purification/detoxification or incineration technique are available for metals/minerals. Therefore, without knowing the purification/detoxification or incineration technique, there is a difficulty to interpret the suitability of the techniques towards safety studies of herbo-minerals.

## 4. Conclusion

In the present study, an attempt was done to collect the commonly used traditional herbo-mineral formulations and their purification/detoxification or incineration techniques. The review illustrated different types of purification/detoxification or incineration techniques of minerals used in herbo-mineral preparations. In addition, there is an urgent need for comprehensive survey or evaluation to check whether purification/detoxification or incineration techniques of minerals are practiced properly in the country.

## Figures and Tables

**Table 1:** Traditional herbo-mineral formulations in Sri Lanka.

	Traditional preparation	Minerals/chemicals in nomenclature	Medicinal plants in number
1	Buddharaja kalka	Cinnabar (Hydragyri sulphidum HgS)-sadilingam,	16
		Trisufide of arsenic (As_2_S_3_)-haritala (orpiment)	
		Rock alum-galmada	
		Asbestos-galnahara	
		Copper sulphate-palmanikkam	
		Arsenic sulfide-*α*-As_2_S_2_ manosila	
		Sodium chloride/rock salt-saindhava lavana	

2	Desandun kalka	Sodium chloride/rock salt-saindhava lavana	7

3	Diyatarama	Copper sulphate-palmanikkam	31 + ghee, bees' honey, neem oil (oil of *Azadirachta indica*)
		Aluminum sulfate [Al_2_(SO_4_)_3_]-sinakkaram	
		Borax powder (sodium borate)-puskara/tankana	
		Ammonium chloride-navasaran	
		Zinc oxide-ridithththam	

4	Koladavundaya	Borax powder (sodium borate)-puskara/tankana	33
		Copper sulphate-palmanikkam	
		Sodium chloride/rock salt-saindhava lavana arsenic sulfide (*α*-As_2_S_2_)-Manashila	
		Aluminum sulfate-sinakkaram	

5	Mandam guliya	Sodium chloride/rock salt-saindhava lavana	22
		Aluminum sulfate [Al_2_(SO_4_)_3_]-sinakkaram	
		Borax powder (sodium borate)-puskara/tankana	

6	Mattupaha	Arsenic sulfide (*α*-As_2_S_2_)-manashila	28
		Trisulfide of arsenic (As_2_S_3_)-haritala (orpiment)	
		Copper sulphate-palmanikkam	
		Borax powder (sodium biborate)-puskara/tankana	
		Aluminum sulfate [Al_2_(SO_4_)_3_]-sinakkaram	
		Rock alum-galmada	
		Asbestos-galnahara	
		Ammonium chloride-navasaran	

7	Ratnadi guliya	Arsenic trioxide, vitrious arsenic, arsenolite (As_2_O_3_) (white arsenic)-gouripashana	7
		Cinnabar (hydragyri sulphidum HgS)-sadilingam	

8	Sanni gajankushaya	Murcury-rasadiya	7
		Borax powder (sodium biborate)-puskara/tankana	
		Sodium chloride/rock salt-saindhava lavana	
		Arsenic trioxide vitrious arsenic, arsenolite (As_2_O_3_) (white arsenic)-gouripashana	

9	Seetarama guliya	Arsenic sulfide-*α*-As_2_S_2_-manosila	29
		Sodium chloride/rock salt-saindhava lavana	
		Copper sulphate-palmanikkam	
		Aluminum sulfate-sinakkaram	
		Borax powder (sodium biborate)-puskara/tankana	
		Rock alum-galmada	
		Trisulfide of arsenic (As_2_S_3_)-haritala (orpiment)	
		Cinnabar (hydragyri sulphidum HgS)-sadilingam	

10	Suranvidura	Borax powder (sodium biborate)-puskara/tankana	23
		Aluminum sulfate-sinakkaram	
		Copper sulphate-palmanikkam	
		Sulpher sublimatum-gendagam	
		Trisulfide of arsenic (As_2_S_3_)-haritala (orpiment)	
		Sodium chloride/rock salt-saindhava lavana	
		China clay-siwanguru	

**Table 2:** Medicinal uses of arsenicals.

Name of the arsenicals	Medicinal uses	References
Realgar	Skin diseases, diarrhea, high fever, coma, heart stroke, abdominal pain, ulcers in tongue and mouth, sore throat, toothache	[38, 39]
Orpiment	Skin diseases, it increases appetite and cures leprosy	[39]
White arsenic	Hemicrania, headache, sinusitis, syphilis, elephantiasis, anemia, psoriasis, asthma, osteoarthritis, splenomegaly, impotency, cancer	[39, 40]

## Data Availability

Data are available on request to the corresponding author.
